# Editorial for the Special Issue, ‘Secondary Metabolites from Microorganisms or Microorganism–Host Interaction?’

**DOI:** 10.3390/biology12121515

**Published:** 2023-12-12

**Authors:** Fengli Zhang, Michel Denis

**Affiliations:** 1 State Key Laboratory of Microbial Metabolism, School of Life Sciences & Biotechnology, Shanghai Jiao Tong University, Shanghai 200240, China; 2 Mediterranean Institute of Oceanography UM 110, Aix-Marseille University, Toulon University, CNRS, IRD, CEDEX 09, 13288 Marseille, France

In this Special Issue, there are 13 published papers from over 10 countries. This Special Issue describes the microorganism–host interaction and symbiotic microorganisms’ secondary metabolites, such as pathogenic factors and antibiotics. This Special Issue provides insights for analyzing the mechanism of the relationship between microorganisms and the host.

The symbiotic relationship between microorganisms and their hosts is ubiquitous and can be mutualistic, commensal, or pathogenic to the host. The microbiota community from microorganisms associated with the gills of fish *Oryzias melastigma* has changed with the enriched osmosensing and metabolic pathways [1]. Research progress in antibiotics from insect-associated actinobacteria has been reviewed, and the molecular ecology of insect–actinobacterial interactions has been mediated by antibiotics [2]. The additional mechanism of pathogen plant colonization by *Streptomyces scabiei* has been introduced [3]. The chemical defense strategy of the sterol *Stentor polymorphus* against *Coleps hirtus* has been reported. The isolated and identified sterols are essential for chemical defense in *S. polymorphus* [4]. Motility, stress, and virulence from *Escherichia coli* associated with tomato fruits have been investigated; this paper provides new information on the physiological adaptation of bacteria colonizing the tomato fruit pericarp [5]. 

Secondary metabolites, such as natural compounds produced by microorganisms, are antibiotics, pigments, toxins, cycle inhibitors, metallophores, enzyme inhibitors, etc. The pigment echinenone (β-carotene pigment) was identified from *Micrococcus lylae* MW407006, pigment production was optimized, and biological activities were investigated. The synthesized nano-echinenone was proven to be safe, and this research paves the way for its potential use in the pharmaceutical industry [6]. *Burkholderia pseudomallei* can cause human neurological melioidosis. Though the bacteria and its pathogenic mechanism remain unknown, the research results indicate that the virulence factor (Cycle-inhibiting factor (Cif)) is involved in the *B. pseudomallei* invasion of human neuronal cells. This paper helps to discover new therapeutic targets for the disease [7]. The novel natural compound, Bacillamide F, isolated from marine *Bacillus atrophaeus* C89 and associated with marine sponge *Dysidea avara*, showed inhibitory activity of acute leukemia cell lines [8]. Important characteristics, including the biosynthesis process, secretion, uptake after metal chelation, and the genetic regulation of three types of metallophores from the human pathogen *Pseudomonas aeruginosa*, are discussed and reviewed [9]. The biosynthesis, export and import processes, and genetic regulation of several metallophores from *Staphylococcus aureus* are reviewed [10]. The structures, biosynthesis pathways, and genetic regulation of metallophore Yersiniabactin and Yersinopine from pathogen *Yersinia pestis* are also reviewed [11]. In archaea *Haloferax mediterranei*, the function of the gene-encoding enzyme involved in the biosynthesis of secondary metabolites peptides is investigated [12]. The potential production of secondary metabolites from the *Streptomyces vinaceusdrappus* strain AC-40 is analyzed via genome mining [13]. 

We would like to thank all the authors for their submissions to this Special Issue. We also thank all the reviewers for dedicating their time and helping ensure the quality of the submitted papers, and, last but not least, the staff at the editorial office of *Biology* for their invaluable assistance.
